# Development of a Comprehensive Gene Signature Linking Hypoxia, Glycolysis, Lactylation, and Metabolomic Insights in Gastric Cancer through the Integration of Bulk and Single-Cell RNA-Seq Data

**DOI:** 10.3390/biomedicines11112948

**Published:** 2023-11-01

**Authors:** Xiangqian Zhang, Yun Li, Yongheng Chen

**Affiliations:** 1 NHC Key Laboratory of Cancer Proteomics & State Local Joint Engineering Laboratroy for Anticancer Drugs, Department of Oncology, Xiangya Hospital, Central South University, Changsha 410008, China; 2 National Clinical Research Center for Geriatric Disorders, Xiangya Hospital, Central South University, Changsha 410008, China

**Keywords:** stomach adenocarcinoma, gastric cancer, hypoxia-related genes, glycolysis-related genes, lactylation-related genes, prognostic model, drug responsiveness, tumor immune microenvironment, immunotherapy

## Abstract

Background: Hypoxia and anaerobic glycolysis are cancer hallmarks and sources of the metabolite lactate. Intriguingly, lactate-induced protein lactylation is considered a novel epigenetic mechanism that predisposes cells toward a malignant state. However, the significance of comprehensive hypoxia–glycolysis–lactylation-related genes (HGLRGs) in cancer is unclear. We aimed to construct a model centered around HGLRGs for predicting survival, metabolic features, drug responsiveness, and immune response in gastric cancer. Methods: The integration of bulk and single-cell RNA-Seq data was achieved using data obtained from the TCGA and GEO databases to analyze HGLRG expression patterns. A HGLRG risk-score model was developed based on univariate Cox regression and a LASSO-Cox regression model and subsequently validated. Additionally, the relationships between the identified HGLRG signature and multiple metabolites, drug sensitivity and various cell clusters were explored. Results: Thirteen genes were identified as constituting the HGLRG signature. Using this signature, we established predictive models, including HGLRG risk scores and nomogram and Cox regression models. The stratification of patients into high- and low-risk groups based on HGLRG risk scores showed a better prognosis in the latter. The high-risk group displayed increased sensitivity to cytotoxic drugs and targeted inhibitors. The expression of the HGLRG *BGN* displayed a strong correlation with amino acids and lipid metabolites. Notably, a significant difference in immune infiltration, such as that of M1 macrophages and CD8 T cells, was correlated with the HGLRG signature. The abundant *DUSP1* within the mesenchymal components was highlighted by single-cell transcriptomics. Conclusion: The innovative HGLRG signature demonstrates efficacy in predicting survival and providing a practical clinical model for gastric cancer. The HGLRG signature reflects the internal metabolism, drug responsiveness, and immune microenvironment components of gastric cancer and is expected to boost patients’ response to targeted therapy and immunotherapy.

## 1. Introduction

Stomach adenocarcinoma (STAD) is a primary type of gastric cancer (GC); it is the sixth most common and the fifth most lethal malignancy worldwide [1]. Despite early interventions, such as addressing high-risk factors including *Helicobacter pylori* infection, smoking, and high nitrite diets, along with routine gastroscopy screenings and timely management of precancerous lesions, the global incidence of GC remains high [2,3]. The expressions of HER2 and CD274, the high microsatellite instability, and the high mutation burdens make immunotherapy a viable treatment option [4]. The clinical trial KEYNOTE-158 has demonstrated the effectiveness of pembrolizumab in managing metastatic or unresectable microsatellite instability/mismatch repair-deficient solid tumors, including GC tumors [5]. However, treatment challenges persist due to drug resistance and poor immune response stemming from cancer heterogeneity. Therefore, developing an effective prognostic model and identifying drug-sensitive individuals are needed to enhance precision medicine for GC.

Dysfunctional cellular metabolic processes, such as enhanced nutrient uptake and accumulated oncometabolites, are distinctive alterations in malignant cells and are essential to tumor progression. Nutrient metabolic processes in tumors include aerobic glycolysis, increased lipid synthesis, and increased demand for amino acids and other nutrients [6]. Metabolites from these nutrient metabolic processes, in turn, affect tumor tissues and organs via reprogramming to regulate gene expression. They act as intercellular signals that mediate cellular communication and guide protein modifications [7]. Metabolomics-based analysis not only facilitates the metabolic profiling of cancers but also provides insights into the survival patterns of tumors. Hypoxia and anaerobic glycolysis are characteristics of rapidly proliferating tumor cells and can lead to an excessive amount of the metabolite lactate, which is a marker of disrupted cellular metabolism [8,9]. Lactate shifts metabolic reprogramming, shapes the acidic tumor microenvironment, and recruits immune cells [10]. Moreover, lactate serves as a donor for the lactylation of proteins. Lactylation is a novel modification that drastically affects cell fate and bridges metabolic reprogramming and epigenetics [11]. For example, histone lactylation, like other epigenetic modifications, regulates the late phase of M1 macrophage polarization [12]. Nonhistone lactylation has also been found to be involved in tumor progression. For example, lactylation of NUSAP1 maintains its stability and constitutes the NUSAP1-LDHA-glycolysis-lactate feedforward loop, thereby linking the Warburg effect to metastases of pancreatic ductal adenocarcinoma [13]. Yang et al. reported elevated lactylation levels in tumor tissues compared to corresponding adjacent tissues, and these high lactylation levels were associated with poor prognosis in GC patients [14]. Furthermore, lactylation-related gene expression profiles can predict prognosis and immune evasion in multiple malignancies [15,16]. However, the connection between the expression profiles of hypoxia–glycolysis–lactylation-related genes (HGLRGs), prognosis, and the tumor microenvironment in patients with GC remains unknown.

**Figure 1:**
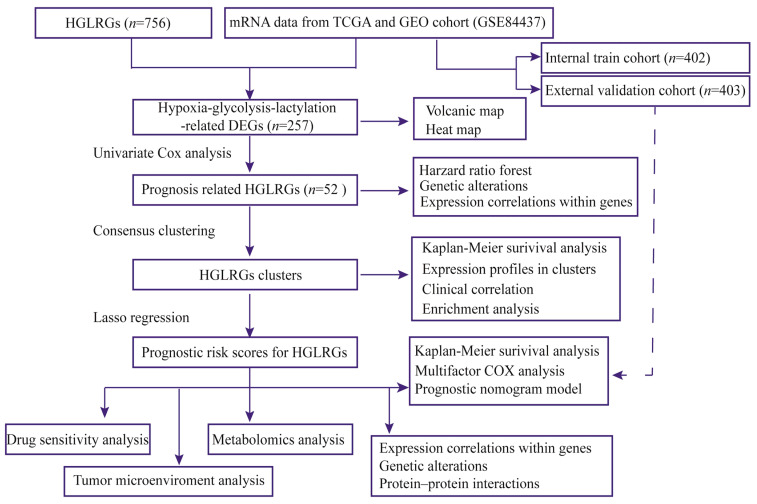
Flow chart for constructing the HGLRG prognostic signature for gastric cancer. Abbreviation: DEGs, differentially expressed genes; GEO, Gene Expression Omnibus; HGLRGs, hypoxia–glycolysis–lactylation-related genes; TCGA, The Cancer Genome Atlas.

In this study, we explored the potential enrichment pathways of different HGLRG patterns and established a risk-score model based on the HGLRG expression profile to predict patient prognosis using The Cancer Genome Atlas (TCGA) and Gene Expression Omnibus (GEO) databases. The obtained risk scores served as a crucial parameter in the development of the nomogram and Cox regression models, which effectively predicted patient survival. Furthermore, we analyzed the responsiveness of patients with different HGLRG risk scores to drug therapy, thereby offering evidence-based guidance for GC treatment (Figure 1). Intriguingly, HGLRG risk scores correlated with immune evasion, suggesting that the identified HGLRG signature could serve as a reference for predicting immunotherapy response among GC patients.

## 2. Materials and Methods

### 2.1. Acquisition of Stomach Carcinoma Data

The mRNA matrix data and related clinical data for STAD were retrieved from the TCGA database [17] and the GSE84437 dataset obtained from the GEO database [18]. Immunohistochemical data of gastric tissues were derived from the online THPA database [19]. Hypoxia–glycolysis–lactylation-related gene sets were comprehensively adopted from previously published studies [16] and the Molecular Signature Database [20], as detailed in Supplementary File S1. The amalgamated cases were randomly allocated to a training cohort and a validation cohort at a 1:1 ratio. The entire research procedure is visually represented in Figure 1.

### 2.2. Identification and Characteristics of HGLRG Molecular Subtypes

The expression levels of HGLRGs were graphically represented using a volcano plot constructed using the R package “limma (Version 3.52.4)”. Following the criteria of FDR < 0.05 and |log2 FC| ≥ 1, differentially expressed genes (DEGs) associated with hypoxia, glycolysis, and lactylation were plotted onto a heatmap. By employing univariate Cox analysis, prognostic HGLRGs in STAD were screened using the criterion of *p* < 0.05. Subsequently, these prognostic DEGs were stratified into two clusters via consensus clustering using “BiocManager (Version 1.30.20)”, with each gene’s expression level analyzed relative to clinical characteristics by employing the “limma (Version 3.52.4)” and “pheatmap (Version 1.0.12)” packages. To uncover the potential functionality of HGLRG patterns, functional enrichment analysis, comprising gene set enrichment analysis (GSEA) and gene set variation analysis (GSVA), was conducted using the R packages “limma (Version 3.52.4)”, “BiocManager (Version 1.30.20)”, “GSEABase (Version 1.58.0)”, and “GSVA (Version 1.44.5)”.

### 2.3. Development and Validation of a Prognostic Signature Based on HGLRGs

To construct an HGLRG-based scoring system indicative of STAD prognosis, LASSO Cox regression was performed with the training group utilizing the “glmnet (Version 4.1.7)” and “survival (Version 3.5.5)” R packages. The risk scores were obtained by multiplying the expression value of each identified risk gene by its specific gene coefficient within the training group, with the gene coefficient being dependent on the optimal penalty parameter λ. The receiver operating characteristic (ROC) curves of the training cohort were generated using the “timeROC (Version 0.4)” R package and served to evaluate the sensitivity and specificity of the scoring system. Furthermore, Kaplan–Meier analysis was used to investigate the discrepancies in overall survival (OS) time between the two risk groups. A nomogram model based on the HGLRG-based scoring system and clinical parameters was generated using the “survival (Version 3.5.5)” and “rms (Version 6.3.0)” packages to predict outcomes. To assess the accuracy and clinical utility of the nomogram model, calibration curves at 1, 3, and 5 years were created using the “survival (Version 3.5.5)” and “rms (Version 6.3.0)” packages; a cumulative hazard curve was constructed using the “survminer (Version 0.4.9)” package; and decision curve analysis was conducted using the “survival (Version 3.5.5)” and “survminer (Version 0.4.9)” packages. The same validation of the scoring system in the total and validation groups was processed at the same time.

### 2.4. Relationship between HGLRG Risk Score and Drug Sensitivity

The drug strategies for STAD mainly involve traditional chemotherapy, small-molecule inhibitors, and immunotherapy. The “oncopredict” R software (Version 4.3.2)” package was employed to examine the differential sensitivity to chemotherapy and small-molecule inhibitors between the high-risk and low-risk patient cohorts. Simultaneously, the “limma (Version 3.52.4)” and “ggpubr (Version 0.4.0)” R packages were utilized to appraise their differential response to immunotherapy.

### 2.5. Relationship between HGLRG Risk Score and the Tumor Immune Microenvironment

The assessment of tumor immunity involves evaluating key factors, such as stroma scores, tumor immune scores, and the abundance of different infiltrated immune cells within the tumor. Additionally, the expression of immune checkpoints and biomarkers of immune cell activation is considered. The tumor microenvironment evaluation was conducted using the R packages “ggpubr (Version 0.4.0)” and “reshape2 (Version 1.4.4)”. Associations between the distribution of immune cells and individual risk scores were explored using the R package “limma (Version 3.52.4)”.

### 2.6. Single-Cell Transcriptome Analysis

Integrative single-cell transcriptomics was analyzed using the GSE134520 and GSE167297 datasets from the GEO database. To ensure data quality, we performed data filtering and quality control using the “Seurat (Version 4.2.0)” R package. Specifically, we obtained probe annotation information and mapped the probes to genes, while removing any multiple matches. For genes with multiple matching probes, we retained the median expression value as the gene expression level. After preprocessing, we applied the filtering criteria to ensure reliable cell subpopulations. Cells expressing each gene in at least 3 cells and containing at least 250 genes per cell were retained for further analysis. Additionally, cells with approximately 30% mitochondrial genes and 100 unique molecular identifiers per cell were included to maintain data integrity. The preprocessed data were then subjected to log normalization, and highly variable genes were identified using the Find Variable Features function. To reduce dimensionality and identify cellular subgroups, principal component analysis was performed. Subsequently, cell clustering was performed using the FindNeighbors and FindClusters functions with the resolution parameter set to 0.1, thus allowing the identification of distinct subgroups with detailed cell annotation.

### 2.7. Statistical Analysis

All statistical analyses were conducted using the R software (version 4.2.2). Survival analysis was performed using Kaplan–Meier curves and univariate and multivariate Cox regression analyses. A two-sided *p* < 0.05 was considered statistically significant and was marked with an asterisk (*).

## 3. Results

### 3.1. Identification of Prognosis-Related Hypoxia–Glycolysis–Lactylation-Related Genes

**Figure 2:**
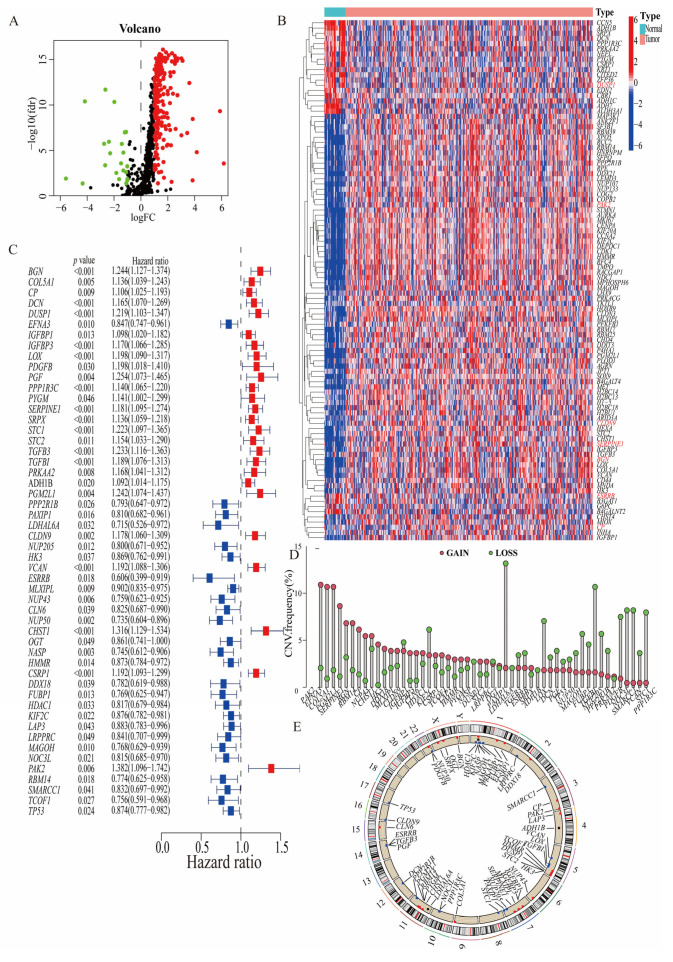
Screening for prognostic HGLRGs. (**A**) A total of 257 DEGs of HGLRGs were identified from the STAD dataset of the TCGA and the GSE84437 dataset. Upregulated genes are marked in red and downregulated genes in green. (**B**) Heatmap showing the expression patterns of the top 50 DEGs. Genes marked in red font represent prognostic DEGs. (**C**) Forest plot of 52 HGLRGs and their hazard ratios used to assess their prognostic significance, as determined based on univariate Cox regression analysis. Risk genes with a hazard ratio greater than 1 are colored red, while protective genes are displayed in blue. Copy number variations (**D**) and chromosome region (**E**) of the 52 DEGs of HGLRGs. Abbreviations: DEGs, differentially expressed genes; HGLRGs, hypoxia–glycolysis–lactylation-related genes.

To explore the expression and distribution characteristics of HGLRGs in GC, we used a cohort of 372 STAD cases from the TCGA database and the GSE84437 dataset containing 483 GC patients. Through an mRNA expression analysis of 756 HGLRGs obtained from GC patients, we identified 235 upregulated and 22 downregulated DEGs (Figure 2A). The top 50 DEGs are depicted in the heatmap (Figure 2B). Additionally, we performed univariate analysis and obtained 52 HGLRGs associated with OS (Figure 2C and Supplementary Table S1). The relationship between the expression levels of these 52 HGLRGs and survival outcomes was analyzed, and the results are shown in Supplementary Figure S1. A copy number variation analysis of these 52 DEGs revealed that *PAK2*, *EFNA3*, and *COL5A1* primarily exhibited a gain of copy number variation, while *PAXIP1* and *NUP205* showed a loss of copy number variation (Figure 2D). The copy number variation mutations of these genes in the chromosome region are displayed in Figure 2E. Furthermore, the associations among these 52 HGLRGs are shown in the prognosis network diagram (Supplementary Figure S2). These results demonstrate that HGLRGs are differentially expressed in GC cancer tissues compared to adjacent normal tissues and are correlated with the prognosis of GC patients.

### 3.2. Consistency Clustering and Pathway Enrichment Analysis among 52 Prognostically Relevant HGLRGs

**Figure 3:**
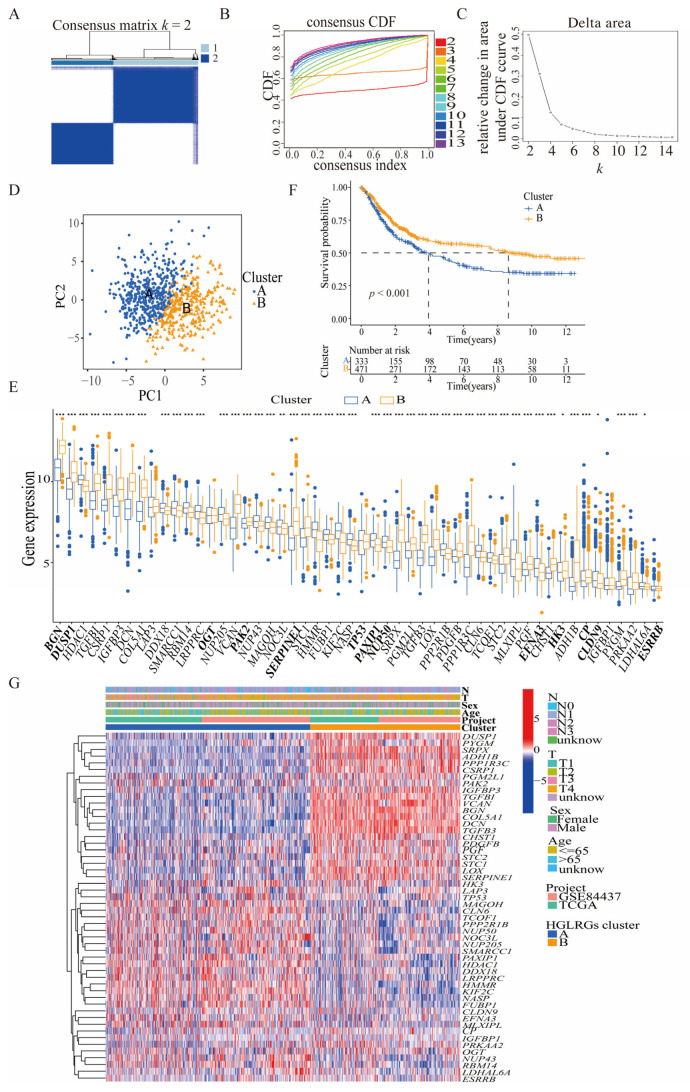
Identification of two HGLRG clusters in gastric cancer patients. (**A**–**C**) Consensus matrix for *k* = 2 was obtained by applying consensus clustering. (**D**) Principal component analysis of patients with STAD. (**E**) Expression levels of 52 HGLRGs in the two clusters. The final 13 HGLRGs are highlighted in bold. (**F**) Overall survival of the two clusters according to Kaplan–Meier curves. (**G**) Heatmap showing the associations between clinicopathological features and the expression profiles of 52 HGLRGs in the two clusters. For (**E**), *, *p* < 0.05; **, *p* < 0.01, ***, and *p* < 0.001.

To further clarify the expression pattern of HGLRGs in GC, a consensus clustering algorithm was utilized to identify two distinct patterns of gene expression among GC patients (Figure 3A–C). The principal component analysis further confirmed the significant differences in gene expression profiles between these two clusters (Figure 3D,E). The Kaplan–Meier survival analysis revealed that patients in Cluster A had significantly shorter OS than those in Cluster B (Figure 3F). A heatmap was constructed to illustrate the associations among the HGLRG clusters, their expression levels, and clinical characteristics (Figure 3G). While the expression of HGLRGs varied between the two clusters, there were no significant differences in clinical characteristics, including patient age group, sex, T and N stages of tumors, and database origin.

**Figure 4:**
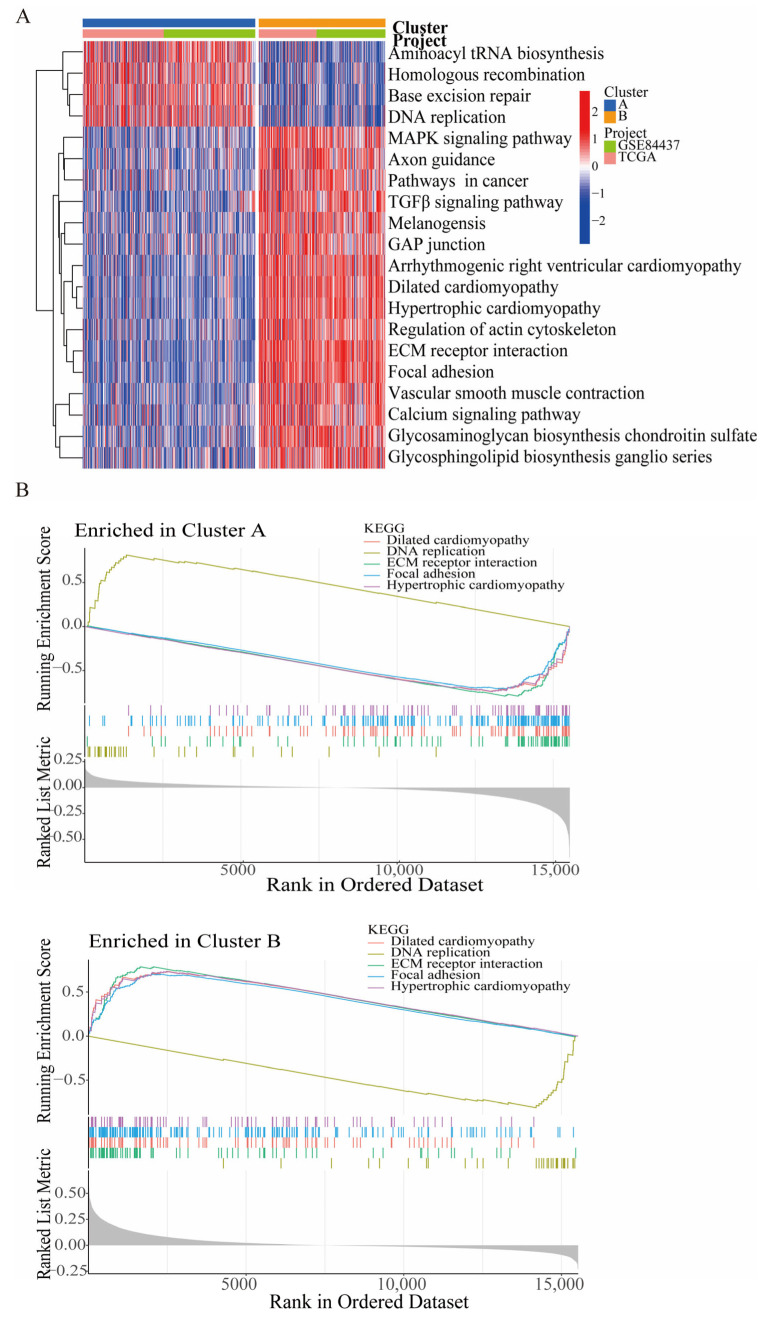
Functional analysis of the two HGLRG clusters. GSVA (**A**) and GSEA (**B**) of pathway enrichment in the two differential HGLRG clusters.

To elucidate the biological functions of the DEGs in these two clusters, GSVA enrichment analysis was conducted and revealed that Cluster A was mainly enriched in tRNA biosynthesis and DNA repair and replication, whereas Cluster B showed enrichment in the MAPK signaling pathway, the pathways in cancer, the TGFβ signaling pathway, melanogenesis, axon guidance, gap junction, and arrhythmogenic right ventricular cardiomyopathy (Figure 4A). In parallel, GSEA (Figure 4B) demonstrated that Cluster A was positively enriched in DNA replication but negatively enriched in ECM receptor interaction, focal adhesion, dilated cardiomyopathy, and hypertrophic cardiomyopathy. However, the GSEA results for Cluster B were opposite to those of Cluster A. Briefly, the clustering of the identified HGLRGs distinguished two distinct GC patterns with differential gene expression profiles and potential functional roles.

### 3.3. Prognostic Significance and Predictive Efficacy of HGLRG-Based Models in GC Survival

**Figure 5:**
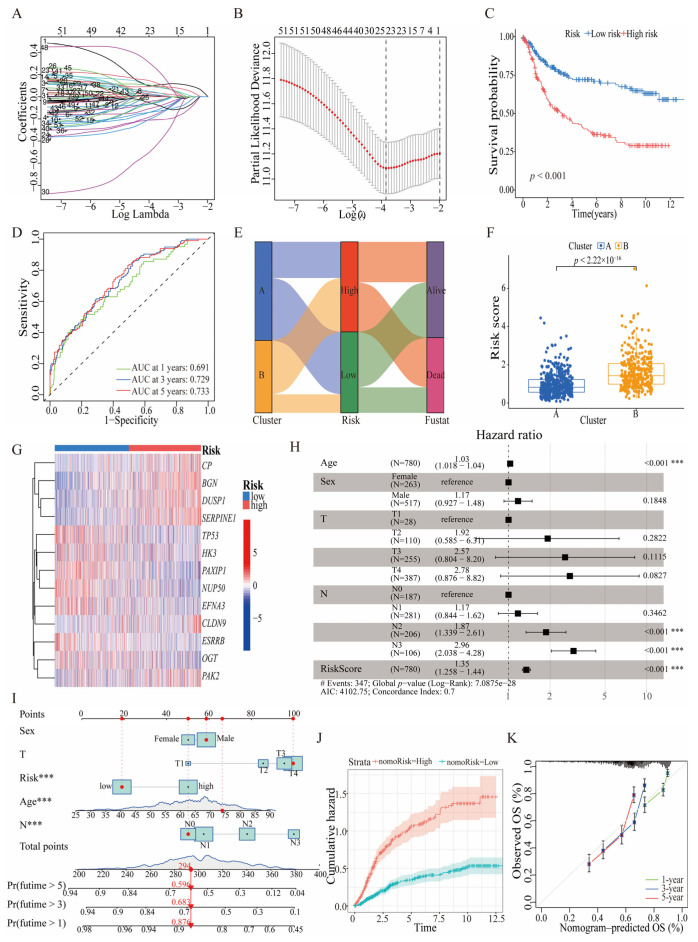
Constructing and validating the HGLRG models in STAD patients. (**A**) The Lasso coefficient profiles illustrate the regression coefficients of the models. (**B**) The partial likelihood deviance was plotted against log (λ) to assess model fitting. (**C**) Kaplan–Meier curves display the overall survival difference between the two risk groups. (**D**) ROC curves demonstrate the predictive efficacy of HGLRG risk scores in STAD patients. (**E**) An alluvial diagram depicts the associations among HGLRG clusters, risk scores, and living status. (**F**) The risk scores of HGLRGs were compared between the two clusters. (**G**) The expression profile of the HGLRG signature is shown for the high- and low-risk groups. (**H**) A comprehensive Cox multivariate regression was used to predict survival probability, incorporating clinicopathological parameters and risk scores. (**I**) The prognostic nomogram model includes the HGLRG signature and clinicopathological indicators. (**J**) Survival probability over time was evaluated using a cumulative hazard curve. (**K**) Calibration curves show the predictive efficacy of the nomogram model at 1-, 3-, and 5-year intervals. For (**H,I**), *** indicates *p* < 0.001.

To construct a quantitative risk-score model containing HGLRGs, we randomly divided all GC patients into a test group and a validation group. Utilizing the optimal value of λ (Figure 5A) and the lowest partial likelihood deviance (Figure 5B), we developed a LASSO-Cox regression model with thirteen HGLRGs. The incorporated genes and their corresponding coefficients are listed in Supplementary Table S2. Based on the risk scores, patients with GC were categorized into high-risk and low-risk groups. The Kaplan–Meier survival curves indicated that patients in the high-risk group had a lower survival probability than those in the low-risk group; these results were observed in the overall GC patient cohort (Figure 5C), in the training group (Supplementary Figure S3A), and in the validation group (Supplementary Figure S3B). The ROC curves effectively predicted patient survival at 1, 3, and 5 years, showing the considerable accuracy of the HGLRG model in predicting survival for all patients (Figure 5D), those in the training group (Supplementary Figure S3C), and those in the validation group (Supplementary Figure S3D). An alluvial diagram was used to integrate the associations among the risk scores, expression clusters, and survival outcomes of patients with GC (Figure 5E). The overall HGLRG risk score reflected a relatively lower score in patients in Cluster A than those in Cluster B (Figure 5F). The details of the differences between the clusters and their HGLRG risk scores are shown in Supplementary Table S3. Collectively, the HGLRG signature effectively reflected the survival status of patients.

The expression profiles of HGLRGs in the two risk groups revealed that the expression levels of *CP*, *BGN*, *DUSP1*, *SERPINE1*, *CLDN9,* and *PAK2* were significantly increased in the high-risk group, while those of *TP53*, *HK3*, *PAXIP1*, *NUP50*, *EFNA3*, *ESRRB*, and *OGT* had relatively low expression in the high-risk group (Figure 5G). Compared with the levels in normal tissues, the expression levels of *DUSP1* and *ESRRB* in STAD tissues were downregulated, and the levels of the other 11 genes were upregulated (Supplementary Figure S4A). Furthermore, we verified the expression of *PAK2* (gene coefficient > 0.5) via immunohistochemistry using the THPA database [19] and found that normal glandular cells were negative for PAK2 expression, but STAD cells were positive for PAK2 expression (Supplementary Figure S4B). The correlations among the expression levels of the thirteen HGLRGs are shown in Supplementary Figure S4C. The locations of the single-nucleotide variants of HGLRGs in STAD patients indicated that TP53 was the most frequently mutated gene (Supplementary Figure S4D). The copy number variation analysis showed that mutations of *OGT*, *BGN*, *PAXIP1*, *CP*, *PAK2*, and *EFNA3* were mostly homologous amplifications, whereas those of *TP53*, *NUP50*, *DUSP1*, *ESRRB*, *HK3*, *CLDN9*, and *OGT* were predominantly homozygous deletions (Supplementary Figure S4E). The protein–protein interaction network obtained from GeneMANIA [21] showed strong physical and genetic interactions among the HGLRGs (Supplementary Figure S4F).

Generally, clinicopathological parameters and tumor grade affect overall survival. We constructed a Cox regression model and a nomogram model to predict OS at 1, 3, and 5 years. The forest plot indicated that age, lymph node stage, and HGLRG score influenced prognosis in the Cox regression model (Figure 5H). The nomogram model showed the same results (Figure 5I). The cumulative hazard curves (Figure 5J) demonstrated a significant increase in the hazard risk of OS over time in the high-risk group compared to the low-risk group. A decision curve analysis was used to assess the nomogram model; the highest clinical benefit was observed for the nomogram model compared to the HGLRG risk model and other clinical parameters in the training, validation, and total cohorts (Supplementary Figure S3E–G). Taken together, the above results demonstrate that these HGLRG-related models are effective in predicting the survival outcomes of patients with STAD.

### 3.4. HGLRG Signature Correlates with Tumor Metabolism

Metabolic reprogramming in tumors facilitates increased nutrient availability to meet the demands of rapid growth. In addition to the well-documented abnormal glycolysis, tumors exhibit uncontrolled amino acid uptake and disrupted lipid metabolism, resulting in distinctive alterations in the amino acid profile within the tumor [6]. We further conducted a comprehensive analysis involving HGLRGs and multiple metabolomic substances. Using a dataset of 62 esophageal and gastric cancer-derived tumor cell lines from the DepMap database [22], we revealed a strong correlation between *BGN* expression and metabolites related to amino acids and lipids. Specifically, *BGN* was positively associated with metabolites such as 2-aminoadipate, glutamine, pyroglutamic acid, dimethylglycine, lysine, serine, and phenylalanine. Conversely, it was negatively correlated with lipid metabolites, such as C36:1 PC, C36:2 PC, C22:0 SM, C54:1 TAG, C36:1 DAG, and C56:2 TAG (Supplementary Figure S5A). Furthermore, our analysis showed statistically significant differences in *BGN* expression concerning 2-aminoadipic acid, glutamine, lysine, and serine (Supplementary Figure S5B–E), while no statistically significant difference was observed for phenylalanine (Supplementary Figure S5F). These findings underscore the intricate relationship between *BGN* expression and metabolic pathways associated with amino acids and lipids, shedding light on the potential significance of *BGN* in the metabolic reprogramming of GC.

### 3.5. HGLRG Signature Predicts Drug Sensitivity in GC

**Figure 6:**
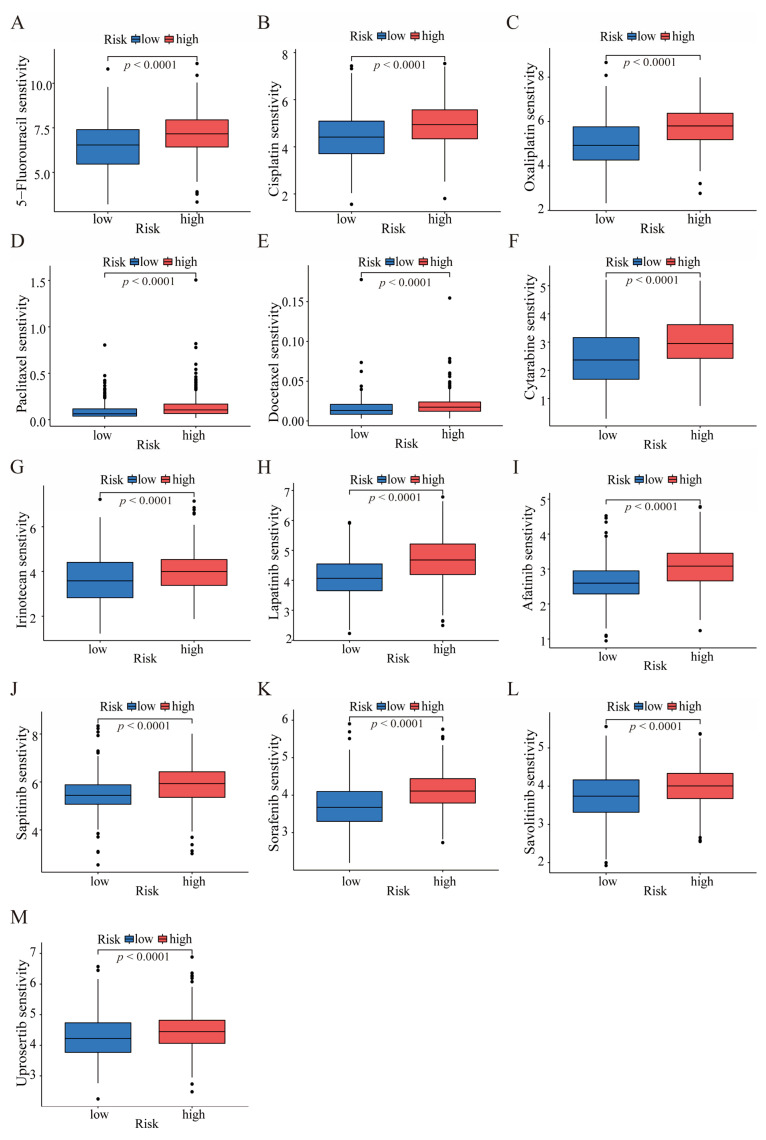
Evaluation of anti-STAD drug responsiveness based on the HGLRG signature. Prediction of drug responsiveness to the cytotoxic agents 5-fluorouracil (**A**), cisplatin (**B**), oxaliplatin (**C**), paclitaxel (**D**), docetaxel (**E**), cytarabine (**F**), and irinotecan (**G**) among high- or low-risk patients. Responsiveness to the HER2 inhibitors lapatinib (**H**) and afatinib (**I**), the EGFR inhibitor sapitinib (**J**), the VEGFR inhibitor sorafenib (**K**), the MET inhibitor savolitinib (**L**), and the AKT inhibitor uprosertib (**M**).

Drug therapy currently plays a crucial role in managing advanced or unresectable GC, as well as nonmetastatic lesions with pathologic T3 and T4 lesions or node-positive GC. Commonly prescribed drugs for GC include cytotoxic agents, such as fluoropyrimidines, platinums, taxanes, and irinotecan, along with small-molecule inhibitors targeting tumor-specific hyperactive kinases or pathways based on tumor molecular features. Consequently, we conducted an analysis of the HGLRG signature and drug sensitivity in GC. The results revealed that patients in the high-risk group demonstrated higher sensitivity to these chemotherapeutic agents (5-fluorouracil, cisplatin, oxaliplatin, paclitaxel, docetaxel, cytarabine, and irinotecan), as well as to inhibitors targeting HER2 (afatinib and lapatinib), the AKT inhibitor uprosertib, the MET inhibitor savolitinib, the VEGFR inhibitor sorafenib, and the EGFR inhibitor sapitinib (Figure 6). These findings provide guidelines for prescribing drugs for GC.

### 3.6. The HGLRG Signature Shapes the Tumor Microenvironment and Predicts Immunotherapy Response in GC

**Figure 7:**
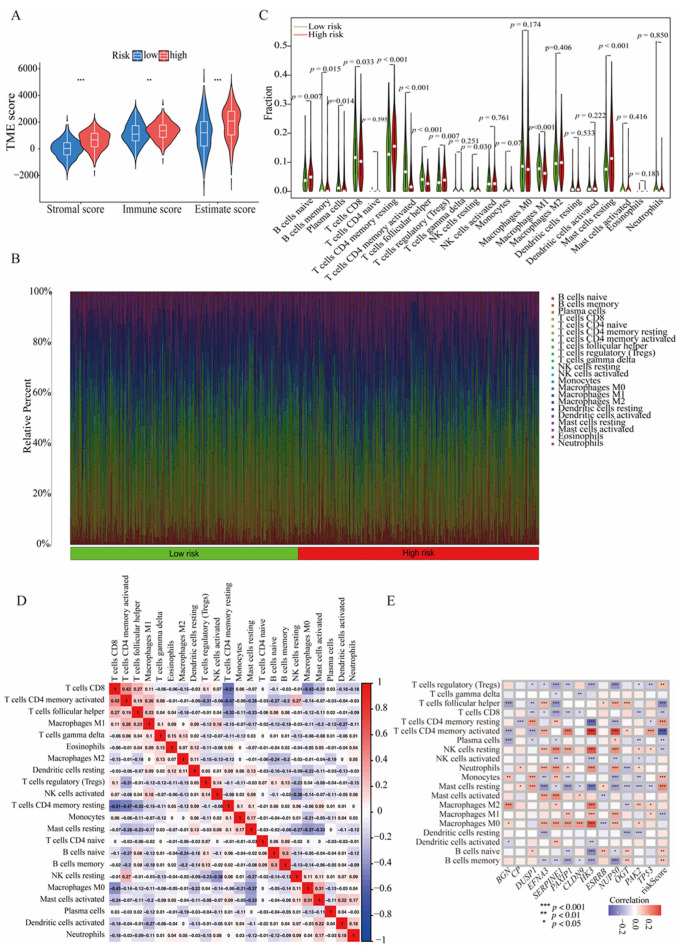
Immune infiltration patterns of the HGLRG signature in gastric cancer. (**A**) Tumor microenvironment scores of the high- and low-risk groups. (**B**) Immune infiltration components in each patient. (**C**) Immune infiltration components in the two risk groups of gastric cancer. (**D**) Correlations among infiltrated immune cells. (**E**) Correlations between immune cells and the HGLRG signature. For (**A**,**C**,**E**), *, *p* < 0.05; **, *p* < 0.01, ***, and *p* < 0.001.

To investigate the relationships among the HGLRG signature, tumor microenvironment, and immunotherapy response in GC, we analyzed the estimated immune scores, abundance of various immune cells, and key immune molecules within the stroma of GC patients. The CIBERSORT algorithm revealed that the high-HGLRG-score group exhibited higher immune scores, stromal scores, and ESTIMATE scores than the low-HGLRG-score group (Figure 7A). The percentage of immune cell infiltration in each case is shown in Figure 7B. Differences in the abundance of each type of immune cells between the two risk groups are shown in Figure 7C; the low-risk group demonstrated a higher abundance of plasma cells, CD8 T cells, activated memory CD4 T cells, and infiltrating M1 macrophages than the high-risk group. A heatmap was constructed to illustrate the correlations among internal immune cells (Figure 7D), and the correlations among model genes, risk scores, and immune cells are shown in Figure 7E. To further assess the potential for immunotherapy, we evaluated the correlations between the HGLRG signature and molecules that suppress T-cell function, including *PDCD1LG2*, *CD274*, *CTLA4*, *LAG3,* and *PDCD1*. We found that *BGN*, *CP*, *DUSP1*, *ESRRB*, *HK3*, *NUP50*, *OGT*, *PAK2*, *PAXIP1*, *SERPINE1*, and *TP53* showed positive associations with these immune molecules, while *CLDN9* and *EFNA3* demonstrated negative associations (Supplementary Figure S6).

### 3.7. HGLRG Signature in GC via Single-Cell Transcriptomics

**Figure 8:**
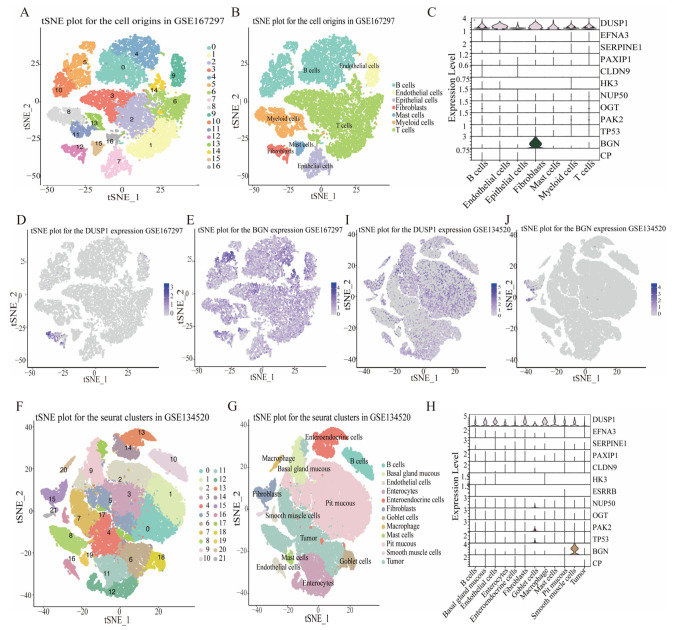
HGLRG signature and immune profiles based on single-cell RNA transcriptomic data. The tSNE analysis revealed distinct cell origins in gastric cancer tissues based on single-cell RNA-seq data from GSE167297 (**A**,**B**) and GSE134520 (**F**,**G**). The expression levels of the HGLRG signature and the percentage of each immune cell in GSE167297 (**C**) and GSE134520 (**H**) were analyzed. The distribution of *DUSP1* (**D**,**I**) and *BGN* (**E**,**J**) is depicted.

Furthermore, single-cell transcriptomics was employed to comprehensively explore the gene expression profiles of specific cellular components in gastric cancer tissues and their dialog. An analysis of GSE67297 revealed 16 cell clusters and 7 medium cell types (Figure 8A,B). *DUSP1* was highly expressed in almost all cells, and *BGN* was mainly expressed in fibroblasts. The remaining genes showed relatively low expression levels in this sample (Figure 8C–E). For GSE34520, 21 cell clusters and 12 cell types were identified (Figure 8F,G). *DUSP1* remained the most abundantly expressed gene in all types of cells, and *BGN* also showed abundant expression in mesenchymal smooth muscle cells (Figure 8H–J). These tumor mesenchymal component analyses suggested that the HGLRG signature was closely related to the tumor microenvironment and could effectively predict immunotherapy response in GC patients.

## 4. Discussion

The Warburg effect is a typical feature of tumor metabolism. It not only provides primary energy and supplies substrates for lactylation to regulate epigenetic gene expression but also influences cell-to-cell communication [8,9,23]. The triad of hypoxia–glycolysis–lactylation interacts in a complex manner, promoting tumor progression and impacting drug responses. Therefore, hypoxia–glycolysis–lactylation not only reflects potential tumor biological mechanisms but also offers targets for cancer therapy. In this study, we identified thirteen hypoxia–glycolysis–lactylation-related genes and provided an HGLRG-related model to predict overall survival. Furthermore, we assessed sensitivity to cytotoxic drugs and targeted agents for GC patients using different risk groups, providing valuable decision making in precision therapy. Additionally, we found significant differences in immune cell abundance among the groups, with more pronounced tumor–immune interactions and increased stromal and immune scores in the high-risk group. Further analysis of single-cell transcriptomic datasets revealed crosstalk between HGLRGs and distinct cell clusters; the abundant expression of *DUSP1* and *BGN* across clusters indicated their critical roles in regulating immune responses. Overall, this potential HGLRG scoring system holds promise for predicting survival outcomes and assists in better assessing patient response to targeted therapies and immunotherapy in GC.

Considering the heterogeneity of GC, single-gene prognostic models have limited value, while machine learning methods incorporating multiple genes offer greater flexibility. We finally developed a prognostic model with thirteen HGLRGs, including *CP*, *BGN*, *DUSP1*, *SERPINE1*, *CLDN9*, *PAK2*, *TP53*, *HK3*, *PAXIP1*, *NUP50*, *EFNA3*, *ESRRB*, and *OGT*. Based on the obtained risk scores, we established a prognosis-related nomogram model and a Cox regression model, with the risk scores significantly contributing to the final model, indicating the vital role of HGLRGs in GC. Previous studies have established prognostic models consisting of only hypoxia-, glycolysis-, or lactylation-related genes signatures in GC. For example, Junyu Huo et al. ultimately screened eight hypoxia-ferroptosis-related genes, including *BGN*, *DUSP1*, *EFNA3*, and *SERPINE1* [24]. Yu et al. identified seven key glycolysis-related genes, such as *CLDN9*, *EFNA3*, and *NUP50* [25]. *EFNA3* and *NUP50* also belong to the lactylation-related signature in GC [15]. The common key genes from different models strongly emphasize the role of HGLRGs in GC prognosis. However, in validating the accuracy of the HGLRG risk model, the ROC curves showed only moderate sensitivity and specificity, which was almost comparable to the accuracy of previously reported hypoxia-related model [26], glycolysis-related model [27], and lactylation-related model [15]. This unsatisfactory result suggests the role of non-metabolic factors in the course of GC and warns of the possible mistakes that can occur when relying on the HGLRG risk model. Thus, better models are needed.

Functional enrichment analysis of HGLRGs showed that Cluster A was mainly involved in DNA damage repair. GC has been identified to show substantial chromosomal instability, microsatellite instability, and genetic mutations, and the tumor suppressor TP53 is the most frequently mutated gene [28,29]. Notably, dysregulated *TP53* is considered a tumorigenic pathway in GC [30]. Studies have shown that *TP53* expression is associated with cancer grade and tissue type, especially in GC with chromosomal instability or microsatellite instability [29,31]. Thus, the overall expression and functional status of *TP53* should be evaluated in GC patients. Similarly, the DNA damage response-related gene *PAXIP1* [32] is elevated not only in GC but also in BRCA1 mutant breast cancers [33]. However, Cluster B was related to tumor signaling pathways, glycosylation biosynthesis, and extracellular matrix. The MAPK pathway was the most prominent signaling pathway enriched in the heatmap. Among the identified HGLRGs, DUSP1 is responsible for inactivating the MAPK pathway through dephosphorylation [34]. OGT, the only enzyme catalyzing O-GlcNAcylation in mammals, increases O-GlcNAcylation during GC carcinogenesis, especially in patients with advanced clinical stage and nodal metastases [35]. O-GlcNAcylation at Reticulon 2 enhances its protein stability and facilitates GC progression [36]. The extracellular matrix component CLDN9 is associated with high invasiveness [37] and mortality [38] in GC. With multiple genes collectively contributing to GC pathogenesis, screening key genes and establishing an appropriate multiple-gene model offer new insights for targeted therapy.

Significant metabolic alterations, such as increased glucose and amino acid (notably glutamine) demand and abnormal lipid synthesis, are key to the ability of cancer cells to sustain survival and rapidly proliferate. In this study, we established a correlation between *BGN* and various metabolites in GC cell lines. *BGN* exhibited a positive association with amino acid-related substrates while displaying a negative correlation with components of lipid metabolism, which is consistent with the energy metabolism features of malignant tumors. BGN is implicated in lipid-related metabolic diseases, such as hypercholesterolemia and atherosclerosis. BGN levels are elevated in dyslipidemic mice and have been validated in hypercholesterolemic patients [39]. As a highly glycosylated extracellular matrix component, BGN exacerbates atherosclerosis via lipid retention through the direct interaction of negatively charged surface components (sulfate and carboxylic acid groups) with positively charged amino acids on lipoproteins [40,41]. Secreted BGN in tumors may act with lipid-related molecules in the microenvironment, thereby regulating lipid metabolism. Yoon et al. observed an increase in glutaminase and resistance to glutaminolysis inhibition in chemotherapy-resistant GC patients. This type of GC improves glucose metabolism as a survival strategy under conditions of starvation, highlighting intricate crosstalk between metabolic pathways [42].

The tumor microenvironment is a complex ecosystem of noncancer components and their metabolites, which plays a critical role in determining cancer outcomes; it performs both tumor suppressor and promoter functions [43]. An analysis of the GC tumor microenvironment revealed significant differences between the two risk groups of HGLRGs, as evidenced by immune cell abundance and immune scores. Analyses of immunotherapy biomarkers and HGLRGs reflected the immune microenvironment profile in GC, showing promise in predicting immunotherapy response. Subsequent in-depth analysis of single-cell transcriptomic datasets highlighted fibroblasts as the primary component in the mesenchyme, potentially implicating them in tumor growth and invasion [44]. Blocking mesenchymal cellular communication might be a promising therapeutic target for GC treatment. Additionally, *DUSP1* and *BGN* emerged as the most abundantly expressed genes among the thirteen HGLRGs in the tumor microenvironment. DUSP1, a critical negative regulator of the immune response, inactivates the MAPK signaling pathway [34]. It also mediates the expression of inflammatory and anti-inflammatory factors through its impact on the activity of the mRNA-stabilizing protein tristetraprolin [34]. BGN is secreted by various cellular components, such as tumor cells, mesenchymal fibroblasts, and macrophages, and triggers inflammation via the TLR-2/4 receptor [45]. Secreted BGN, together with HGF and SPP1, has emerged as a crucial factor responsible for recruiting suppressive myeloid cells in prostate cancer [46]. A comprehensive analysis of bulk and single-cell profiling datasets from pan-cancer cohorts indicated that *BGN* serves as a significant risk indicator of OS and immunotherapy responsiveness. This conclusion is supported by the evidence that patients with elevated *BGN* levels tend to exhibit lower infiltration of CD8^+^ T cells and a higher likelihood of poor treatment response than those with lower BGN levels [47]. However, the roles of abundant *DUSP1* and *BGN* in the GC microenvironment still need to be further investigated.

Notably, an advantage of our study is that it is the first integrated analysis of HGLRGs in the clinical practice to assess the prediction outcomes and personal optimal drug prescription; additionally, it reveals the relationship between HGLRGs and their internal metabolites within tumors. While bioinformatics research based on public transcriptomics databases provides valuable insights, it has inherent limitations, such as limited sample size and source diversity. As verified by the ROC curves, the accuracy of the HGLRG signature is moderate; thus, more biomarkers beyond mRNAs need to be incorporated to improve the model fit in the future. To enhance the applicability of predictive models, more diverse samples from different ethnicities and populations are needed for calibration. Moreover, the gap between gene transcription and actual protein expression levels, as well as the internal factors affecting protein function, requires the incorporation of multiple omics studies, such as proteomics. Considering tumor heterogeneity and evolution, combining single-cell analyses with spatiotemporal genomics can provide a more comprehensive understanding of the dynamic changes in tumors. Finally, further in vitro and in vivo experiments are necessary to elucidate the internal associations and specific roles of these key genes in GC.

## 5. Conclusions

In summary, the HGLRG signature could effectively categorize GC patients into different risk groups. A gene model comprising thirteen HGLRGs could be used to predict the survival prognosis of GC patients. The HGLRG signature reveals internal connections in the metabolism of tumors, and this signature correlates with drug responsiveness and the tumor immune microenvironment, thus being able to guide personalized treatment strategies for GC patients.

## Data Availability

Data availability statements are available after contact with the corresponding author: yonghenc@163.com.

## Supplementary Materials

The following supporting information can be downloaded at: https://www.mdpi.com/article/10.3390/biomedicines11112948/s1.

## Abbreviations

AKT, Akt kinase; AMPK, adenosine 5‘-monophosphate-activated protein kinase; BGN, biglycan; CD274, CD274 molecule; CLDN9, claudin 9; COL5A1, collagen type V alpha 1 chain; CP, ceruloplasmin; CTLA4, cytotoxic T-lymphocyte-associated protein 4; DEGs, differentially expressed genes; DUSP1, dual-specificity protein phosphatase 1; ECM, extracellular matrix; EFNA3, ephrin A3; EGFR, epidermal growth factor receptor; ESRRB, estrogen-related receptor beta; FC, fold change; FDR, false discovery rate; GC, gastric cancer; GEO, Gene Expression Omnibus; GSEA, gene set enrichment analysis; GSVA, gene set variation analysis; HER2, human epidermal growth factor receptor 2; HGF, hepatocyte growth factor; HGLRGs, hypoxia–glycolysis–lactylation-related genes; HK3, hexokinase 3; LAG3, lymphocyte-activating 3; LASSO, least absolute shrinkage and selection operator; LDHA, lactate dehydrogenase A; MAPK, mitogen-activated protein kinase; MET, MET proto-oncogene receptor tyrosine kinase; NUP205, nucleoporin 205; NUP50, nucleoporin 50; NUSAP1, nucleolar and spindle-associated protein 1; OGT, O-linked N-acetylglucosamine transferase; OS, overall survival; PAK2, p21 (RAC1)-activated kinase 2; PAXIP1, paired box interacting protein 1; PDCD1, programmed cell death 1; PDCD1LG2, programmed cell death 1 ligand 2; ROC, receiver operating characteristic; SERPINE1, serpin family E member 1; SPP1, secreted phosphoprotein 1; STAD, stomach adenocarcinoma; T, tumor; TCGA, The Cancer Genome Atlas; TGFβ, transforming growth factor-β; TLR, Toll-like receptor; TP53, tumor protein p53; VEGFR, vascular endothelial growth factor receptor.
