# Supplementary material for: Development of a Comprehensive Gene Signature Linking Hypoxia, Glycolysis, Lactylation, and Metabolomic Insights in Gastric Cancer through the Integration of Bulk and Single-Cell RNA-Seq Data
Source: Biomedicines. 2023 Nov 1;11(11):2948. doi: 10.3390/biomedicines11112948 (PMC10669360; doi:10.3390/biomedicines11112948)
Supplement: Supplementary file 1 [file biomedicines-11-02948-s001.zip › Supplementary Figures and Tables .pdf]

Development of a Comprehensive Gene Signature Linking Hypoxia, Glycolysis, Lactylation, and Metabolomic Insights in Gastric Cancer through the Integration of Bulk and Single-Cell RNA-Seq Data

Supplementary Figures and Tables

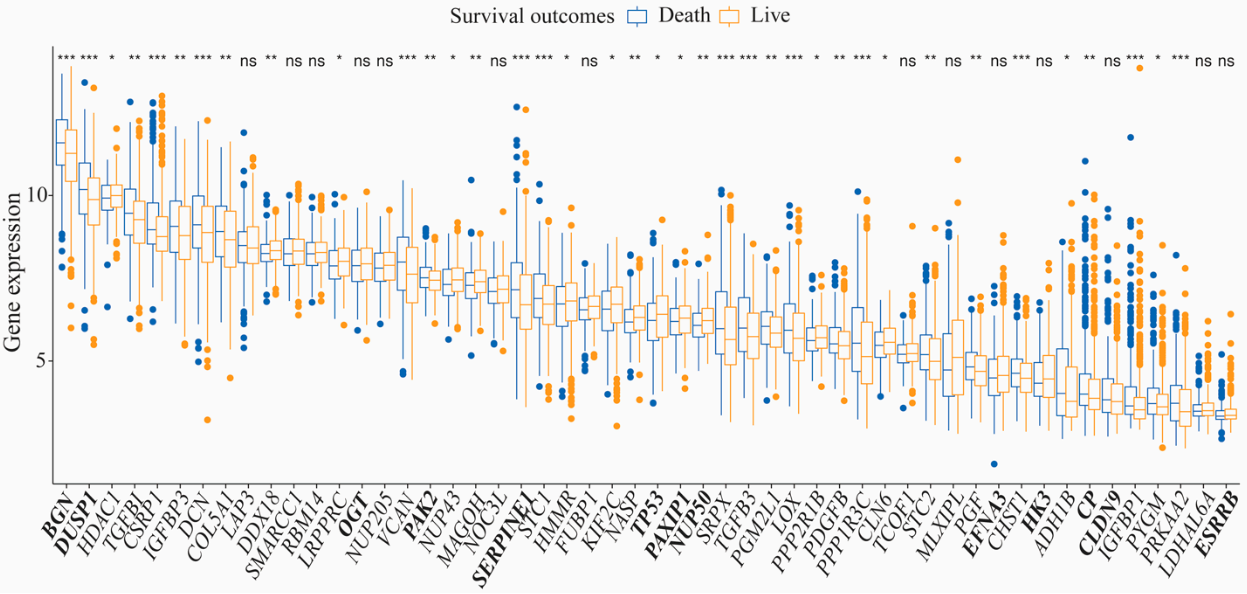

Supplementary Figure S1. Expression levels of 52 HGLRGs and survival outcomes in gastric cancer.

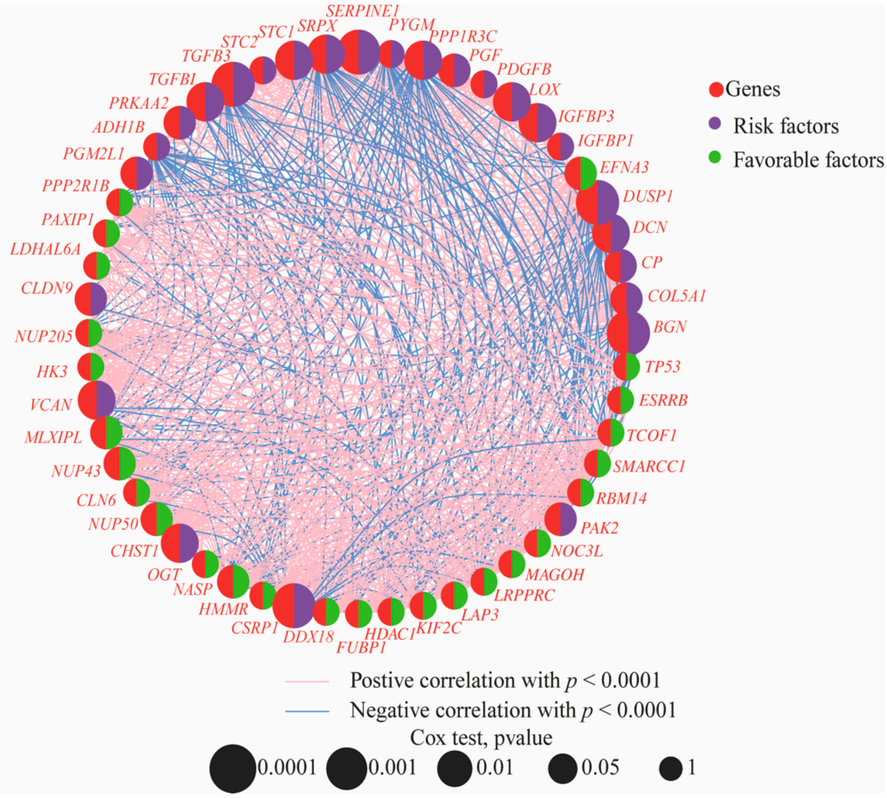

Supplementary Figure S2. Network diagram showing the positive and negative correlations between the 52 HGLRGs.

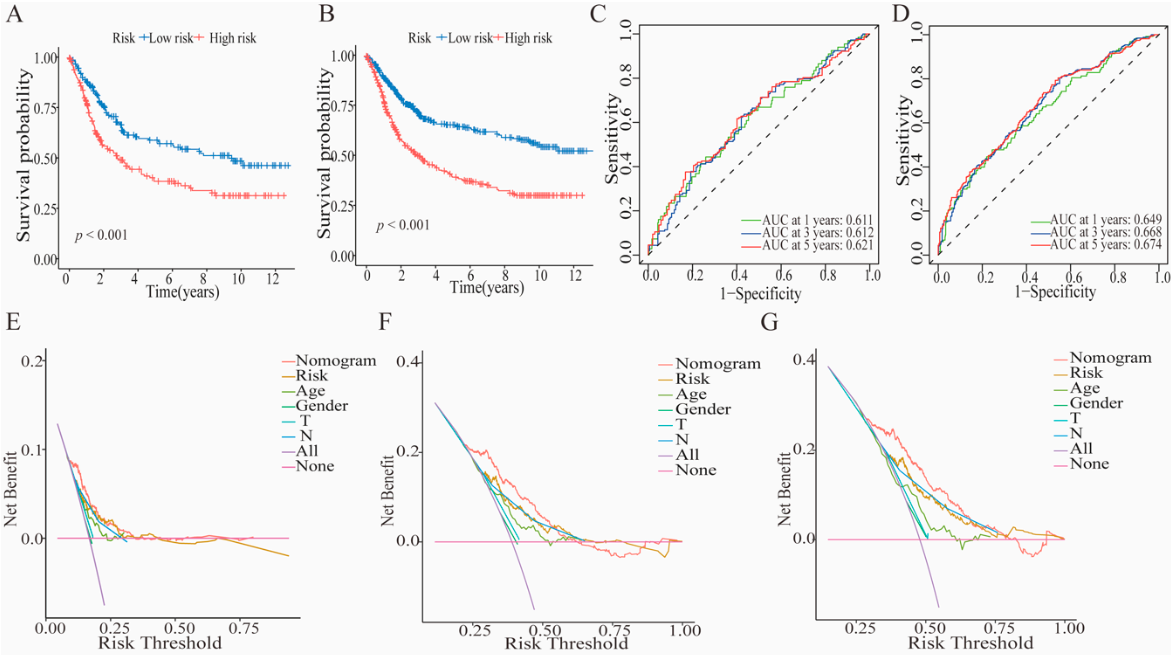

Supplementary Figure S3. Evaluating HGLRG prognostic models in gastric cancer.

The Lasso Cox regression model of gastric cancer demonstrated that the risk score was correlated with overall survival in both the training cohort (A) and validation cohort (B). ROC curves illustrating the predictive efficacy of the HGLRG risk score in the training cohort (C) and validation cohort (D). Decision curve analysis showed that the nomogram model based on the HGLRG signature provided the best clinical benefit.

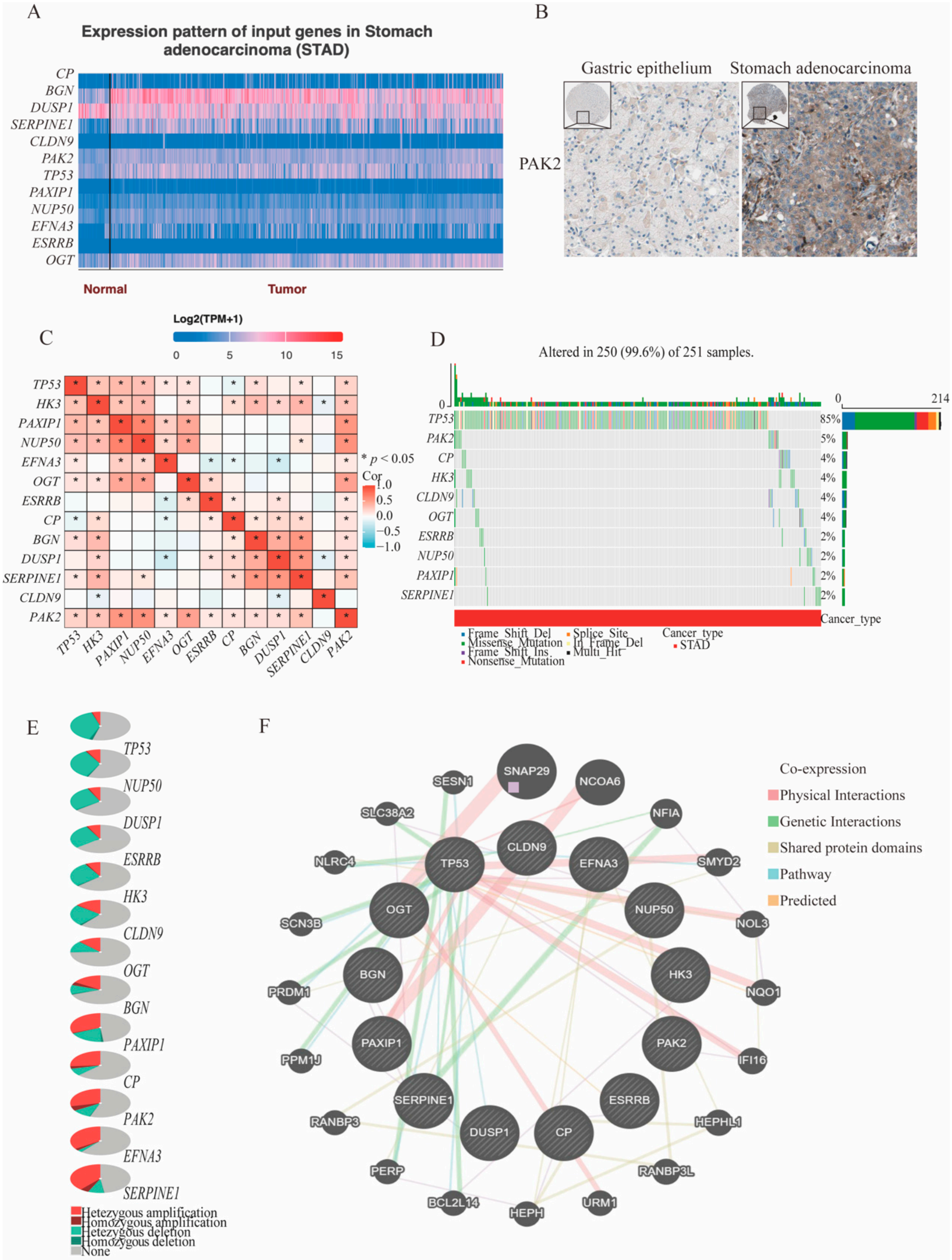

Supplementary Figure S4. Expression levels, epigenetic variations, and protein–protein interactions of HGLRGs in gastric cancer.

(A) The heatmap displays the mRNA expression levels of HGLRGs in gastric cancer. (B) Representative immunohistochemistry images showing PAK2 expression in normal stomach and gastric cancer tissues from the THPA database. (C) Gene correlation analysis revealed the relationships among prognostic HGLRGs. (D) The distribution of the top 10 single nucleotide variants of HGLRGs in gastric cancer was depicted based on the GSCA database. (E) Pie charts illustrating the CNVs in HGLRGs in gastric cancer using the GSCA database. (F) Protein–protein interactions among intersectional proteins are displayed. *, of statistical significance.

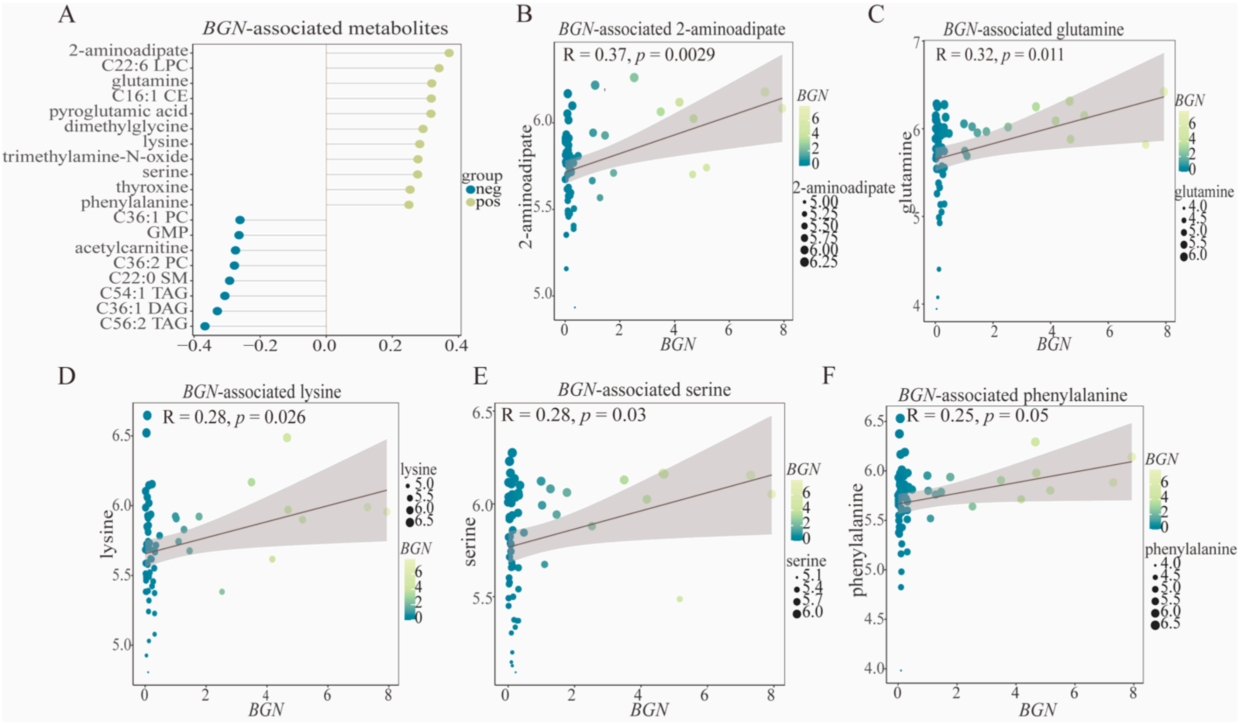

Supplementary Figure S5. BGN and tumor metabolites.

(A) Bar graph showing the correlation between the abundance of BGN expression and metabolites within the tumor cells. (B-F) Scatter plots illustrating the correlation between BGN expression and 2-aminoadipate, glutamine, lysine, serine, and phenylalanine.

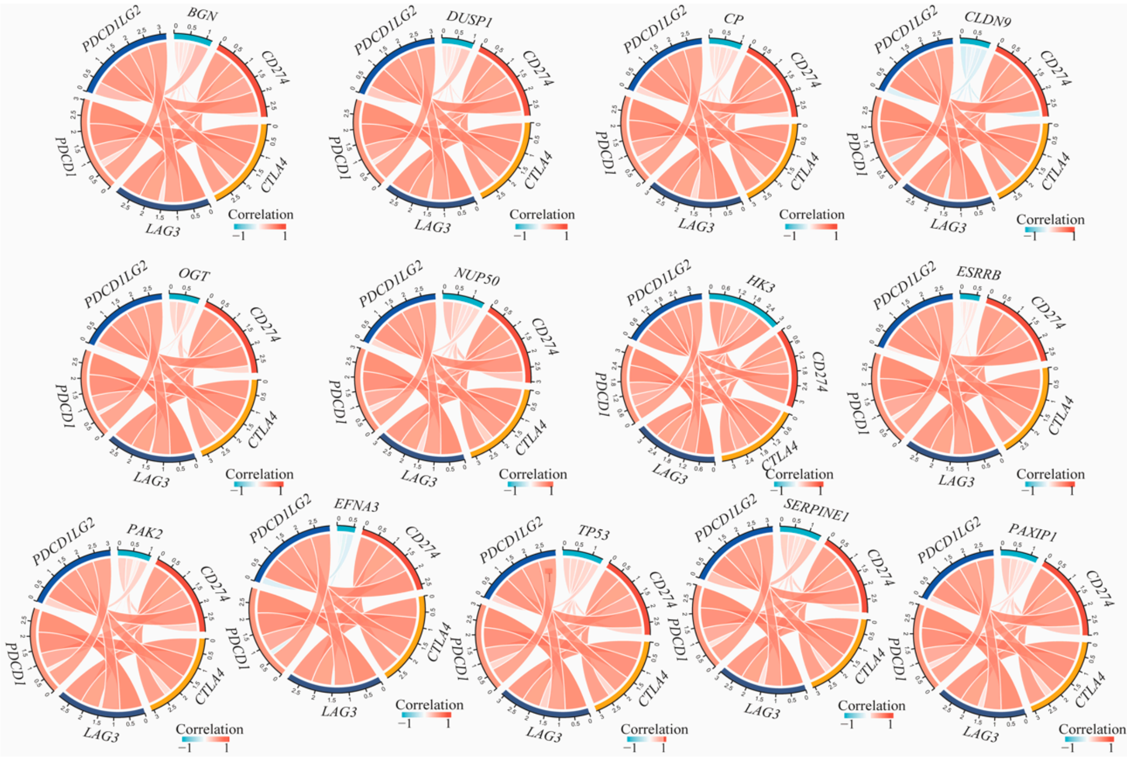

Supplementary Figure S6. Correlation of immune checkpoint molecules with the HGLRG signature.

Supplementary Table S1: Correlation between expression levels of 52 HGLRGs in adjacent normal and cancer tissues and prognosis

Gene	Expression levels				Prognosis value			
	conMean	treatMean	log FC	*p*	HR	HR.95L	HR.95H	*p*
ADH1B	26.806	6.807	-1.977	<0.001	1.092	1.014	1.175	0.020
**BGN**	78.897	676.313	3.100	<0.001	1.244	1.127	1.374	<0.001
CHST1	0.904	4.099	2.181	<0.001	1.316	1.129	1.534	<0.001
**CLDN9**	0.303	4.379	3.855	<0.001	1.178	1.060	1.309	0.002
CLN6	3.888	12.062	1.634	<0.001	0.825	0.687	0.990	0.039
COL5A1	28.941	109.545	1.920	<0.001	1.136	1.039	1.243	0.005
**CP**	2.281	11.093	2.282	0.025	1.106	1.025	1.193	0.009
CSRP1	348.079	113.037	-1.623	0.012	1.192	1.093	1.299	<0.001
DCN	165.066	81.751	-1.014	<0.001	1.165	1.070	1.269	<0.001
DDX18	13.795	34.234	1.311	<0.001	0.782	0.619	0.988	0.039
**DUSP1**	750.008	303.558	-1.305	<0.001	1.219	1.103	1.347	<0.001
**EFNA3**	4.688	13.199	1.493	<0.001	0.847	0.747	0.961	0.010
**ESRRB**	0.612	0.287	-1.089	0.036	0.606	0.399	0.919	0.018
FUBP1	17.403	40.472	1.218	<0.001	0.769	0.625	0.947	0.013
HDAC1	54.363	118.095	1.119	<0.001	0.817	0.679	0.984	0.033
**HK3**	0.854	3.408	1.997	<0.001	0.869	0.762	0.991	0.037
HMMR	2.882	17.903	2.635	<0.001	0.873	0.784	0.972	0.014
IGFBP1	0.074	4.397	5.884	<0.001	1.098	1.020	1.182	0.013
IGFBP3	40.249	159.995	1.991	<0.001	1.170	1.066	1.285	0.001
KIF2C	4.127	24.719	2.583	<0.001	0.876	0.782	0.981	0.022
LAP3	42.636	98.388	1.206	<0.001	0.883	0.783	0.996	0.043
LDHAL6A	0.213	0.514	1.268	<0.001	0.715	0.526	0.972	0.032
LOX	4.865	22.507	2.210	<0.001	1.198	1.090	1.317	<0.001

LRPPRC	24.196	59.642	1.302	<0.001	0.841	0.707	0.999	0.049
MAGOH	26.897	54.257	1.012	<0.001	0.768	0.629	0.939	0.010
MLXIPL	3.235	15.256	2.238	<0.001	0.902	0.835	0.975	0.009
NASP	10.609	27.753	1.387	<0.001	0.745	0.612	0.906	0.003
NOC3L	5.470	14.272	1.384	<0.001	0.815	0.685	0.970	0.021
NUP205	13.086	32.566	1.315	<0.001	0.800	0.671	0.952	0.012
NUP43	10.311	25.175	1.288	<0.001	0.759	0.623	0.925	0.006
**NUP50**	13.164	28.125	1.095	<0.001	0.735	0.604	0.896	0.002
**OGT**	18.128	45.844	1.339	<0.001	0.861	0.741	1.000	0.049
**PAK2**	34.035	69.367	1.027	<0.001	1.382	1.096	1.742	0.006
**PAXIP1**	3.043	7.349	1.272	<0.001	0.810	0.682	0.961	0.016
PDGFB	8.194	21.216	1.373	<0.001	1.198	1.018	1.410	0.030
PGF	1.761	5.924	1.750	<0.001	1.254	1.073	1.465	0.004
PGM2L1	2.060	10.258	2.316	<0.001	1.242	1.074	1.437	0.004
PPP1R3C	44.482	8.542	-2.380	<0.001	1.140	1.065	1.220	<0.001
PPP2R1B	9.269	19.175	1.049	<0.001	0.793	0.647	0.972	0.026
PRKAA2	8.031	2.883	-1.478	<0.001	1.168	1.041	1.312	0.008
PYGM	15.763	2.361	-2.739	<0.001	1.141	1.002	1.299	0.046
RBM14	15.958	32.533	1.028	<0.001	0.774	0.625	0.958	0.018
**SERPINE1**	17.025	111.989	2.718	<0.001	1.181	1.095	1.274	<0.001
SMARCC1	23.271	55.757	1.261	<0.001	0.832	0.697	0.992	0.041
SRPX	34.486	16.028	-1.105	<0.001	1.136	1.059	1.218	<0.001
STC1	10.762	31.522	1.550	<0.001	1.223	1.097	1.365	<0.001
STC2	1.150	5.134	2.159	<0.001	1.154	1.033	1.290	0.011
TCOF1	8.110	22.968	1.502	<0.001	0.756	0.591	0.968	0.027
TGFB3	4.301	8.715	1.019	0.001	1.233	1.116	1.363	<0.001

TGFBI	42.087	89.628	1.091	<0.001	1.189	1.076	1.313	0.001
**TP53**	17.760	43.451	1.291	<0.001	0.874	0.777	0.982	0.024
VCAN	7.859	41.038	2.384	<0.001	1.192	1.088	1.306	<0.001

The final 13 HGLRGs are highlighted in bold. conMean, mean expression in normal tissues; FC, fold change; HR, hazard ratio; treatMean, mean expression in gastric cancer.

Supplementary Table S2: LASSO Cox regression model of included HGLRGs

Genes	Coefficients
BGN	0.142
CP	0.160
DUSP1	0.155
EFNA3	-0.180
SERPINE1	0.122
PAXIP1	-0.323
CLDN9	0.194
HK3	-0.284
ESRRB	-0.837
NUP50	-0.269
OGT	-0.286
PAK2	0.508
TP53	-0.207
