# Supplementary material for: Development of a Comprehensive Gene Signature Linking Hypoxia, Glycolysis, Lactylation, and Metabolomic Insights in Gastric Cancer through the Integration of Bulk and Single-Cell RNA-Seq Data
Source: Biomedicines. 2023 Nov 1;11(11):2948. doi: 10.3390/biomedicines11112948 (PMC10669360; doi:10.3390/biomedicines11112948)
Supplement: Supplementary file 1 [file biomedicines-11-02948-s001.zip › Supplementary file 1.pdf]

Hypoxia-glycolysis-lactylation-related gene sets
ACKR3
ADM
ADORA2B
AK4
AKAP12
ALDOA
ALDOB
ALDOC
AMPD3
ANGPTL4
ANKZF1
ANXA2
ATF3
ATP7A
B3GALT6
B4GALNT2
BCAN
BCL2
BGN
BHLHE40
BNIP3L
BRS3
BTG1
CA12
CASP6
CAV1
CAVIN1
CAVIN3
CCN1
CCN2
CCN5
CCNG2
CDKN1A
CDKN1B
CDKN1C
CHST2
CHST3
CITED2
COL5A1
CP
CSRP2
CXCR4
DCN
DDIT3
DDIT4
DPYSL4
DTNA
DUSP1
EDN2
EFNA1
EFNA3
EGFR
ENO1

ENO2
ENO3
ERO1A
ERRFI1
ETS1
EXT1
F3
FAM162A
FBP1
FOS
FOSL2
FOXO3
GAA
GALK1
GAPDH
GAPDHS
GBE1
GCK
GCNT2
GLRX
GPC1
GPC3
GPC4
GPI
GRHPR
GYS1
HAS1
HDLBP
HEXA
HK1
HK2
HMOX1
HOXB9
HS3ST1
HSPA5
IDS
IER3
IGFBP1
IGFBP3
IL6
ILVBL
INHA
IRS2
ISG20
JMJD6
JUN
KDELR3
KDM3A
KIF5A
KLF6
KLF7
KLHL24
LALBA
LARGE1

LDHA
LDHC
LOX
LXN
MAFF
MAP3K1
MIF
MT1E
MT2A
MXI1
MYH9
NAGK
NCAN
NDRG1
NDST1
NDST2
NEDD4L
NFIL3
NOCT
NR3C1
P4HA1
P4HA2
PAM
PCK1
PDGFB
PDK1
PDK3
PFKFB3
PFKL
PFKP
PGAM2
PGF
PGK1
PGM1
PGM2
PHKG1
PIM1
PKLR
PKP1
PLAC8
PLAUR
PLIN2
PNRC1
PPARGC1A
PPFIA4
PPP1R15A
PPP1R3C
PRDX5
PRKCA
PYGM
RBPJ
RORA
RRAGD
S100A4

SAP30
SCARB1
SDC2
SDC3
SDC4
SELENBP1
SERPINE1
SIAH2
SLC25A1
SLC2A1
SLC2A3
SLC2A5
SLC37A4
SLC6A6
SRPX
STBD1
STC1
STC2
SULT2B1
TES
TGFB3
TGFBI
TGM2
TIPARP
TKTL1
TMEM45A
TNFAIP3
TPBG
TPD52
TPI1
TPST2
UGP2
VEGFA
VHL
VLDLR
WSB1
XPNPEP1
ZFP36
ZNF292
PRKAA2
ADH1B
PGM2L1
CHPF2
TPST1
IL13RA1
SDHC
BIK
KIF20A
MIOX
NUPL2
ARNT
FUT8
PPP2R1B
KIF2A

DSC2
NUP88
SPAG4
PHKA2
PRKAG3
NUP155
PDHB
POM121
GPR87
TGFA
INS
GOT1
B4GALT2
PLOD1
NANP
LCT
PPP2R5D
AKR1A1
GCLC
PAXIP1
LDHAL6A
ANG
AGL
ALDH3A1
CHST6
NDC1
TSTA3
ALDH9A1
GUSB
ADH5
NUP85
G6PC2
PFKM
ADPGK
HS6ST2
NT5E
ADH4
GALK2
STMN1
GYS2
LDHB
PDHA2
PYGL
ALDH2
B4GALT7
PC
GMPPA
ALDH3B1
CD44
CLDN9
NUP205
PRXL2C
PGK2
HK3

NUP133
MPI
DEPDC1
LDHAL6B
NUP37
ALDH3B2
GALE
NUP98
G6PD
POM121C
ADH7
B4GALT4
GCKR
NUP54
TIGAR
SEC13
AAAS
TPR
PDHA1
ME2
NDST3
PKP2
HOMER1
PMM2
POLR3K
GPD1
CHST12
ARPP19
ENTPD5
SEH1L
PGP
DHTKD1
NUP160
MYOG
PPP2CB
PGAM1
RPE
CENPA
ELF3
CDK1
MET
CYB5A
STAT3
RANBP2
AK3
LHPP
NUP107
PFKFB2
ZBTB7A
PRKAG1
VCAN
DLAT
GNE
AC074143.1

GNPDA2
HIF1A
CACNA1H
ALDH1A3
CLDN3
P2RX7
NUP210
MED24
HKDC1
ALDH1B1
ESRRB
ME1
RAE1
EIF6
GALM
INSR
SOD1
RARS
NCOR1
FKBP4
MLXIPL
B3GAT3
ADH6
CBFA2T3
PKM
COG2
GLCE
B4GALT1
BPNT1
PLOD2
FBP2
OGDHL
SLC25A10
GMPPB
NUP153
QSOX1
HS2ST1
PCK2
NUP43
PPARA
G6PC
CHST4
SLC25A13
ACSS2
NUP62
PPIA
PSMC4
IDUA
NSDHL
CAPN5
PRKACA
PRPS1
ACTN3
B3GAT1

CLN6
PPP2CA
NUP35
ALG1
ZBTB20
PPP2R1A
ADH1A
ABCB6
TXN
ACSS1
PGAM4
AURKA
GNPDA1
DLD
NUP214
AGRN
XYLT2
CHPF
BPGM
NUP50
GFPT1
HTR2A
TREX1
MERTK
RBCK1
JMJD8
AC016586.1
IDH1
PRKACB
NUP58
ENO4
LHX9
OGDH
PRKACG
COPB2
PYGB
HDAC4
NDUFV3
B3GNT3
TALDO1
CHST1
IGF1
PFKFB4
GOT2
MDH1
MDH2
PRKAG2
PFKFB1
NUP93
EXT2
PRKAA1
ECD
SOX9
CTH

GAL3ST1
SDC1
HAX1
SLC16A3
OGT
ARTN
ADH1C
ALDH3A2
EGLN3
SLC35A3
AC010618.1
NASP
ALDH7A1
NUP188
SRD5A3
TFF3
HMMR
ACAT2
ACIN1
ADAR
ADNP
AHNAK
ALB
ALDH1A1
ALYREF
ARGLU1
ARID1A
ARID3A
ARID3B
BCLAF1
BOLA2
BRD4
BTF3
BZW1
BZW2
C19orf53
CACYBP
CALD1
CALM1
CALML5
CALR
CBR1
CBX3
CBX5
CCNA2
CCT5
CD2BP2
CDC5L
CDV3
CDV3
CDYL
CEBPZ
CFDP1
CHD4

CHERP
CNN2
CNN3
COPS4
CRABP2
CSRP1
CWC15
DDX17
DDX18
DDX21
DDX39A
DDX39B
DDX3X
DDX41
DDX42
DDX46
DDX5
DECR1
DFFA
DHRS7
DHX16
DHX9
EAF1
ECHDC1
EDF1
EEF1A1
EEF1G
EEF2
EHMT2
EIF3D
EIF3J
EIF4G1
EIF4G2
EIF4H
EMG1
ENSA
FABP5
FAM50A
FKBP3
FLYWCH2
FUBP1
GATAD2A
GATAD2B
GFAP
GIGYF2
GTF2F1
GTF2I
H1-2
H1-3
H1-5
H2AFV
H2AFZ
H2AJ
H2AX

H2AZ1
H2AZ2
H2BC13
H2BC14
H2BC18
H2BC5
H2BU1
H3-3A
H3C1
H3C15
H4C1
HCFC1
HDAC1
HDAC2
HDGF
HDGFL2
HEXIM1
HIST1H1C
HIST1H2BB
HIST1H2BD
HIST1H2BH
HIST1H2BK
HIST1H2BL
HIST1H2BN
HIST1H2BO
HIST1H3A
HIST1H4A
HIST2H2BE
HIST2H2BF
HISTIH2BH
HLTF
HMGA1
HMGB1
HMGN1
HMGN2
HMGN3
HMGN4
HNRNPA1
HNRNPC
HNRNPD
HNRNPF
HNRNPH1
HNRNPK
HNRNPL
HNRNPM
HNRNPU
HSDL2
HSPE1
IARS2
IFI16
IK
IKZF1
ILF2
ILF3

IRF2BP2
JMJD1C
JPT1
JPT2
KHDRBS1
KHSRP
KIF2C
KRT1
KRT10
LAP3
LCP1
LEMD3
LGALS1
LRPPRC
LSP1
MAGOH
MAGOHB
MAP2K4
MAPRE1
MBD2
MBP
MDC1
MKI67
MNDA
MPHOSPH6
MSN
MTA1
MTA2
MYH13
NCDN
NCL
NEFL
NHLRC2
NOC3L
NOLC1
NONO
NOP2
NPM1
NSUN2
NUCKS1
NUDT21
NUDT5
PABPC1
PABPN1
PAK2
PARP1
PCBP1
PCBP2
PCMT1
PCNP
PDAP1
PDLIM1
PES1
PFN1

PHC3
PHF6
PKM2
POLDIP3
PPIL4
PPM1G
PPP1CB
PPP1CC
PPP1R2B
PRAM1
PRCC
PRDX1
PRKDC
PRPF6
PSMA7
PSMC1
PSME3IP1
PTBP1
PTMA
RACGAP1
RALYL
RAN
RB1
RBM10
RBM14
RBM17
RBM25
RBM39
RBMX
RCC2
RECQL
RFC1
RFC4
RIMS1
RPA1
RPL13
RPL14
RPL22
RPL24
RPL29
RPL5
RPS11
RPS23
RPS27A
RRP1B
RSL1D1
S100A11
S100A6
SAFB
SARNP
SATB1
SET
SF3A1
SF3B1

SFPQ
SFPQ
SH3GL1
SMAP
SMARCA5
SMARCC1
SMARCC2
SMC3
SNRPA1
SPR
SPR14
SRP14
SRRM1
SRRM2
SSB
SUB1
SUMO2
TCOF1
TERF2
THOC2
THRAP3
THUMPD1
TKT
TMA7
TMPO
TMSB4X
TOP2B
TP53
TPM4
TRIM28
TRIR
TSSC4
U2AF2
U2SURP
UBE2E1
UBE2M
UPF1
VARS
VIM
WAS
WBP11
WDR33
WIZ
XPO5
XRCC4
YLPM1
ZC3H18
ZC3H4
ZMYM3
ZNF207
ZNF280C
ZNF706
ZNFX1
ZRANB2

ZYX
